# Diagnosis and Management of Autonomic Dysfunction in Dementia Syndromes

**DOI:** 10.1007/s11940-019-0581-2

**Published:** 2019-07-10

**Authors:** Louise M. Allan

**Affiliations:** Institute of Health Research, College of Medicine and Health, University of Exeter, South Cloisters Building, St Luke’s Campus, Heavitree Road, Exeter, EX1 2LU UK

**Keywords:** Autonomic nervous system, Dementia, Orthostatic hypotension, Urinary tract symptoms, Lewy body disease

## Abstract

**Purpose of review:**

Autonomic dysfunction is common in dementia, particularly in the Lewy body dementias. This review considers the evidence for autonomic dysfunction in dementia, common symptoms and potential management options.

**Recent findings:**

Autonomic dysfunction has been shown in Alzheimer’s disease and Lewy body dementias. Common symptoms include orthostatic dizziness, syncope, falls, urinary tract symptoms and constipation. Non-pharmacological management of orthostatic hypotension should include bolus water drinking. Pharmacological management may include the use of midodrine or droxidopa although the latter is not available in Europe. Atomoxetine is a noradrenaline reuptake inhibitor which may be useful if further clinical trials become available. Management of constipation may include the use o f probiotics, osmotic laxatives such as macrogol and chloride type 2 channel activators such as lubiprostone. Management of urinary tract symptoms may include the use of mirabegron.

**Summary:**

There is a dearth of clinical trials for autonomic dysfunction in dementia and most of the evidence is imputed from trials in Parkinson’s disease. However, pragmatic recommendations may be made. There is a need for controlled clinical trials in people with dementia.

## Introduction

The autonomic or “involuntary” nervous system (ANS) is responsible for the control of many bodily functions that we are not usually aware of, such as heart rate, blood pressure, body temperature, saliva and tear production, pupil size, gut motility and sphincter control. It is one of the most fundamental homeostatic mechanisms within mammalian physiology and can be disturbed in many disease processes. There is a reason to believe that the ANS may be affected by dementia as neuropathological lesions can be found in the ANS in many types of dementia. In Alzheimer’s disease (AD), lesions have been found in the insula and anterior cingulate cortex [1]. ANS dysfunction is a common feature of the Lewy body disorders which include Parkinson’s disease dementia (PDD) and dementia with Lewy bodies (DLB) [2•]. Aggregates of misfolded alpha-synuclein are deposited in the neurons, including axons, forming Lewy bodies and Lewy neurites. These are found in the post-synaptic peripheral autonomic neurons. DLB is also associated with underactivity of the cholinergic nervous system [3]. Acetylcholine is essential for parasympathetic and pre-ganglionic sympathetic neurotransmission.

Common symptoms of ANS dysfunction are outlined in Table 1 and include orthostatic dizziness, syncope, falls, urinary tract symptoms and constipation. Orthostatic symptoms are typically worse in the morning, after meals, during a rise in body temperature, with prolonged standing, and with physical activity. Total autonomic symptom scores, urinary symptoms, constipation and postural dizziness have been shown to be significantly higher in PDD, DLB and vascular dementia (VAD) patients than either controls or AD patients and are associated with reduced activities of daily living, depression and poorer quality of life [4]. Falls are a particularly significant symptom as they are associated with the subsequent decline in activities of daily living, institutionalisation and mortality. Orthostatic hypotension has been shown to be a risk factor for falls in dementia [5]. Orthostatic hypotension may be associated with poorer cognitive prognosis in Parkinson’s disease (PD), but whether this is due to causation or association with more severe underlying disease is unclear [6, 7].

**Table 1:** Symptoms of autonomic dysfunction

Symptoms of orthostatic hypotension	Other autonomic symptoms
Light-headedness or dizziness Syncope Falls Weakness Fatigue Head and neck pain (coat hanger pain) Vertigo Pallor Clamminess Blurred vision Palpitations Tremulousness Cognitive difficulties Anxiety	Bloating, nausea and vomiting Dysphagia Dry mouth or sialorrhoea Dry eyes Loss of sweating or excessive sweating Sensitivity to glare Diarrhoea and constipation Urinary frequency, urgency and urge incontinence Urinary retention Erectile dysfunction

A number of studies have examined ANS dysfunction in dementia. The results in AD have been conflicting, with some studies showing evidence of ANS dysfunction [8–12] and some studies showing that some patients may have evidence of autonomic neuropathy but as a group, no deficits in heart rate variability [13•, 14]. A recent meta-analysis of heart rate variability studies in AD also showed conflicting evidence with deficits in the time domain rather than the frequency domain and high heterogeneity of results [15]. VAD may also be associated with some ANS dysfunction, but again the evidence is conflicting [13•, 14, 16, 17]. However, as expected, the most prominent ANS dysfunction has been shown in the Lewy body dementias [13•, 18–21]. The study of orthostatic hypotension (OH) alone may indicate some ANS dysfunctions, and OH has been shown to be more common in all types of dementia, but other factors may also contribute to OH in dementia [13•, 22–24]. Orthostatic hypotension may be asymptomatic in dementia [25].

## Diagnosis

Clinical autonomic function testing usually focuses upon the neurocardiovascular system as this can be investigated non-invasively. Commonly used tests include the measurement of orthostatic changes in blood pressure and heart rate, the cardiovascular responses to the Valsalva manoeuvre (expiration against a closed glottis), respiratory sinus arrhythmia during respiration at a controlled frequency, response to a cold stimulus (cold pressor test) and isometric exercise [26]. Abnormalities in more than 3 of these tests are usually taken as an indication of clinically significant autonomic failure [27]. Alternatively, a composite scoring system may be used which has been shown to correlate with severity of disease and prognosis in other common causes of autonomic neuropathy [28]. In the clinic, a simple measurement of orthostatic blood pressure change is the most commonly used test. Orthostatic hypotension is defined in recent consensus criteria as a sustained reduction of systolic blood pressure of at least 20 mmHg or diastolic blood pressure of 10 mmHg within 3 min of standing or head-up tilt to at least 60° on a tilt table. In patients with supine hypertension, a reduction in systolic blood pressure of 30 mmHg may be a more appropriate criterion for orthostatic hypotension because the magnitude of the orthostatic blood pressure fall is dependent on the baseline blood pressure [29]. An active stand is probably preferable to a tilt test as it reproduces normal activity. It is not clear whether intermittent blood pressure measurement or beat-to-beat measurement is most appropriate for the diagnosis of orthostatic hypotension. The beat-to-beat measurement may detect some transient drops in blood pressure which are of unknown prognostic significance [30]. Baroreflex failure may result in supine hypertension in some patients.

Heart rate variability has been used to detect ANS dysfunction, but this is a research tool and is not useful for the clinical diagnosis of ANS dysfunction. Meta-iodobenzylguanidine (MIBG) is a physiological analogue of norepinephrine, and cardiac ^123^I-MIBG scintigraphy has previously been used as a non-invasive method of screening for local myocardial sympathetic nerve damage in various cardiac and neurological diseases. The fourth consensus report of the diagnostic criteria for DLB recommends that MIBG scanning may be used as a biomarker of DLB [31].

The assessment of dysphagia may be undertaken by oropharyngeal videofluoroscopy and constipation by measurement of stool transit time. Urinary symptoms may be investigated by urodynamic testing which may show neurogenic detrusor overactivity. Other methods of evaluation of autonomic function include quantitative sudomotor axon testing, thermoregulatory sweat testing, sympathetic skin response, microneurography, pupillography, measurement of tear production, nocturnal penile tumescence studies and sphincter electromyography, but these are usually undertaken in specialist centres.

## Current treatment

There are few clinical trials which have examined the management of ANS dysfunction specifically in dementia. Therefore, current management has to be pragmatic and is guided by the evidence for the management of ANS dysfunction in related populations. There is some evidence for the management of OH in older people in general and there is also a body of evidence for the management of ANS dysfunction in Parkinson’s disease which is of particular relevance to the Lewy body dementias.

### Orthostatic hypotension

#### Non-pharmacological interventions

Initial management of OH is non-pharmacological. Other causes of OH besides ANS dysfunction, such as dehydration and medications, should be sought and treated. Hypotensive drugs have been reported to be associated with syncope in dementia [32]. These include diuretics, nitrates, α-blockers and angiotensin-converting enzyme inhibitors. However, the effect of stopping antihypertensives on OH is not necessarily clear. Studies have shown an association between antihypertensives and OH but these have been observational [33]. There is some evidence from a randomised controlled trial of withdrawal of antihypertensives that prevalence of OH was reduced in those who did withdraw the drugs, but intention to treat analyses were not significant [34]. Conversely, the SPRINT trial found a lower prevalence of OH in intensively treated patients, but this did not apply to older groups and people with a standing BP of less than 115 mmHg were excluded from the study [35].

Patients should be given conservative advice about avoiding sudden changes in posture, maintaining their fluid and salt intake to ensure adequate hydration, eating smaller and more frequent meals and avoiding heat stressors such as hot tubs. However, patients should avoid physical inactivity which worsens ANS function due to physical deconditioning. Some experts recommend measuring urinary sodium excretion to determine whether salt intake is adequate and if not to replace salt with either increased dietary intake or salt tablets. Patients who have a 24-h urinary sodium excretion of below 170 mmol can be treated with 1–2-g supplemental sodium three times a day [36]. Elevating the head of the bed has been recommended to reduce nocturia and supine hypertension [36], but a systematic review did not find any evidence that this is effective [37]. Specific non-pharmacological interventions include bolus water drinking, physical counter-manoeuvres (standing cross-legged), lower limb compression and abdominal compression [38]. A recent qualitative study has shown that physical counter-manoeuvres were the most popular non-pharmacological therapy in older people as they are convenient, require no equipment and can be done as required. However, people with dementia may have difficulty learning these manoeuvres. Bolus water drinking (480 ml of room-temperature tap water, to consume as much as possible within 5 min) is also well tolerated and acceptable to patients, but some do have concerns about urinary frequency. Lower limb and abdominal compression were the least acceptable. Stockings may be difficult to get on and aesthetically unpleasing and abdominal binders may be uncomfortable [39]. In terms of efficacy, bolus water drinking has been found to be the most efficacious intervention, followed by abdominal compression and physical counter-manoeuvres. Compression stockings were the least efficacious [40].

#### Pharmacological interventions

If non-pharmacological measures are insufficient, then pharmacological interventions may be required. Midodrine is a prodrug whose metabolite, desglymidodrine, is an agonist of α_1_ adrenoceptors that increase vascular resistance and blood pressure. It may be used in Europe and the USA. It is the only licensed pharmacological treatment for OH in the UK. A systematic review of 7 trials including 325 patients has shown that midodrine increases the standing BP but does not improve postural drop. However, there is some evidence that symptoms are improved. Evidence is of low quality and further trials are required [41]. Treatment is started at 2.5 mg once or twice daily and may be increased up to 10 mg three times daily. The first dose should be taken 1 h before rising and the last dose at least 4 h before bedtime to avoid supine hypertension. Side effects of midodrine include hypertension, scalp tingling and piloerection. Contraindications are liver disease, severe heart disease, acute kidney injury, urinary retention, phaeochromocytoma and thyrotoxicosis.

Droxidopa is a more recent drug for neurogenic OH which has mainly been used in Japan but has now received FDA approval in the USA. It is not licensed in Europe. Droxidopa is a norepinephrine prodrug that is converted into norepinephrine both in the central nervous system and in peripheral tissues, including sympathetic peripheral nerve endings. A meta-analysis of four trials including 494 patients showed that it was effective in reducing dizziness, overall symptoms and difficulty with activity. Droxidopa was also effective in improving standing systolic blood pressure [42•]. Treatment may commence at 100 mg three times daily increasing to 600 mg three times daily. Like midodrine, it should be given during waking hours and avoided before bedtime to avoid supine hypertension. Side effects include headache, dizziness, nausea, fatigue and supine hypertension. Caution is advised in patients with congestive heart failure and chronic renal failure.

Atomoxetine is a noradrenaline reuptake inhibitor which exerts a vasopressor effect and is an emerging therapy for OH. In a randomised controlled trial, atomoxetine produced a greater pressor response in upright systolic blood pressure compared with midodrine [43]. Further clinical trials of this agent are required. The most common side effects are decreased appetite, dry mouth, insomnia and nausea. Severe hepatitis has been anecdotally reported.

Fludrocortisone is often used off-label to treat OH but evidence for its use is low and it is recommended by consensus opinion only [44••]. It acts by increasing renal sodium and water reabsorption, thus expanding intravascular blood volume. There may be long-term effects of increased vascular resistance. Treatment begins at 100 μg once daily increasing to 200 μg once daily. Side effects include supine hypertension, hypokalemia and oedema. Caution is advised in patients with congestive heart failure.

Cholinesterase inhibitors such as pyridostigmine have been suggested as treatments for OH. Most patients with neurodegenerative dementias will already be on cholinesterase inhibitors. Lower rates of OH have been reported in a Cochrane review of rivastigmine treatment for PDD [45] and improved responses to orthostasis in AD have been shown [46]. However, another study has shown worsened heart rate variability in people with dementia treated with donepezil [47].

### Supine hypertension

Supine hypertension in neurogenic OH patients is arbitrarily defined as a systolic blood pressure above 150 mmHg or diastolic pressure above 90 mmHg while in the supine position [44••]. It is common in patients with chronic ANS dysfunction and difficult to manage because its treatment has to be balanced with treatment for OH, with management of one condition often exacerbating the other. Expert recommendations for the management of supine hypertension in the setting of neurogenic OH suggest that supine hypertension requires intervention if systolic blood pressure exceeds the range of 160–180 mmHg, but in patients with the largest drops in orthostatic BP, this may have to be tolerated to avoid symptoms of OH and the increased risk of falls [44••]. Patients with neurogenic OH and supine hypertension should be advised to avoid supine posture during the day and elevate the head of the bed as tolerated during the night. Fludrocortisone should be avoided and short-acting antihypertensives such as captopril 25 mg, hydralazine 10–25 mg or nitroglycerine patch (0.1 mg/h, remove in the morning) may be given at night. Patients should be warned about the risk of falls when getting up at night because of OH; a bedside urinal or commode may be required.

### Dysphagia and sialorrhoea

Patients with dysphagia should be referred to a speech and language therapist. Useful interventions may include postural and behavioural changes such as reduced meal volumes and slow eating. Expiratory muscle strength training (EMST) and video-assisted swallowing therapy (VAST) may be effective dysphagia treatments in PD [48]. A randomised control trial exploring interventions to prevent aspiration including 132 PDD with dysphagia found lower rates of aspiration as evidenced on videofluoroscopy, with honey-thickened fluids [49]. Another study noted improved swallowing function objectively in 48 Lewy body dementia patients referred for videofluoroscopy with carbonated liquids [50]. In patients with severe dementia, it may be appropriate to consider at risk feeding, in which the risk of aspiration is tolerated in order to improve quality of life [51].

Sialorrhoea is often due to reduced swallowing frequency and a portable metronomic brooch to act as a reminder to swallow may help. Local anticholinergics are not suitable for patients with dementia because of their cognitive side effects. A randomised cross-over trial of glycopyrrolate (1 mg, twice or three times a day) in 23 PD patients found that 9 of patients had a clinically relevant improvement in sialorrhoea 75 over a 4-week period [52]. Side effects include dry mouth, urinary retention, constipation and blurred vision. An evidence-based review of botulinum toxin injection to the salivary glands appears effective and safe in PD, although repeat injections are typically required every 3 to 6 months [53].

### Gastroparesis

Dietary modifications, including a low-fat diet with small frequent meals and liquid nutrients, can help with gastroparesis [2•]. D_2_ receptor blockers such as metoclopramide and domperidone have been used but are associated with an increase in the QT interval and risk of arrhythmia. Erythromycin and azithromycin stimulate motilin receptors but adverse events include gastrointestinal toxicity, ototoxicity, antibiotic resistance and QT prolongation. Muscarinic agonists and cholinesterase inhibitors may be used but many patients may already be on cholinesterase inhibitors. Ghrelin receptor agonists are being tested in clinical trials [54].

### Constipation

Non-pharmacological interventions may help with the initial management of constipation. These include increasing the amount of fibre in the diet, increasing fluid intake and increasing physical activity. Medications which may exacerbate constipation such as anticholinergic agents and opiates should be reviewed. One controlled clinical trial found that fermented milk products with probiotics (e.g. kefir) resulted in a higher increase in the number of complete bowel movements in patients with PD [55].

Pharmacological interventions include the use of bulk laxatives such as psyllium and osmotic laxatives such as polyethylene glycol (macrogol). Psyllium has been shown to increase the frequency of bowel movement in PD patients [56]. A randomised controlled trial of macrogol showed an improvement in symptoms of constipation in PD patients [57]. Lubiprostone is a locally acting chloride type 2 channel activator. In a randomised controlled clinical trial of PD patients, a marked or very marked clinical global improvement was reported by 16 of 25 (64.0%) subjects receiving drug vs 5 of 27 (18.5%) subjects receiving placebo [58].

### Urinary tract symptoms

Urinary symptoms in ANS dysfunction may be related to detrusor overactivity or underactivity. Non-pharmacological interventions include increasing fluid intake because concentrated urine may exacerbate detrusor overactivity. Bladder training may help and has been found to be useful in a recent pilot trial in PD, but has not been investigated in dementia and may be more difficult for dementia patients to undertake.

Anti-muscarinic drugs which are usually used to treat detrusor overactivity are better avoided in dementia because of their cognitive side effects. Mirabegron is a selective β3-adrenergic receptor which elicits relaxation of the detrusor muscle during the storage phase and improves bladder capacity. It has been suggested as an option for the management of urinary symptoms in dementia by the National Institute of Health and Clinical Excellence [59]. Treatment can be commenced at 25–50 mg once daily. Side effects include urinary retention, pelvic/abdominal pain and hypertension.

Incomplete bladder emptying is uncommon in dementia but can occur if ANS dysfunction is present. If the post-void residual is greater than 100 ml, then intermittent self-catheterisation can be considered. This may need to be performed by a caregiver. If this is not possible, then an indwelling catheter may have to be considered.

### Sexual dysfunction

Erectile dysfunction, problems with ejaculation and difficulty achieving orgasm are common in ANS dysfunction. Contributing factors such as stress, depression, anxiety and medications should be considered. Diuretics, β-blockers and selective serotonin reuptake inhibitors can all cause erectile dysfunction. Options for management of erectile dysfunction include phosphodiesterase type 5 (PDE-5) inhibitors, intracavernosal injection therapy, vacuum pump devices, intraurethral prostaglandin suppositories and surgical placement of penile prostheses. When prescribing PDE 5 inhibitors, a short-acting agent such as sildenafil should be used because of the risk of hypotension.

### Sweating

Excessive sweating is common in PD patients. There are no treatment trials but there was a consensus from a recent expert Delphi panel group that patients may benefit from the use of loose-fitting clothing, cotton bedding for night sweats and antiperspirants as well as avoidance of triggers, e.g. alcohol, spicy foods and hot rooms [60].
